# Monitoring patient care through health facility exit interviews: an assessment of the Hawthorne effect in a trial of adherence to malaria treatment guidelines in Tanzania

**DOI:** 10.1186/s12879-016-1362-0

**Published:** 2016-02-03

**Authors:** Baptiste Leurent, Hugh Reyburn, Florida Muro, Hilda Mbakilwa, David Schellenberg

**Affiliations:** 1 Department of Infectious Disease Epidemiology, Faculty of Epidemiology and Population Health, London School of Hygiene and Tropical Medicine, London, UK; 2 Department of Disease Control, Faculty of Infectious and Tropical Diseases, London School of Hygiene and Tropical Medicine, London, UK; 3 Joint Malaria Programme, Kilimanjaro Christian Medical Centre, Moshi, Tanzania

**Keywords:** Hawthorne effect, Patient exit interview, Observer effect, Malaria, Rapid diagnostic test, Primary health care facilities, Tanzania

## Abstract

**Background:**

Survey of patients exiting health facilities is a common way to assess consultation practices. It is, however, unclear to what extent health professionals may change their practices when they are aware of such interviews taking place, possibly paying more attention to following recommended practices. This so-called Hawthorne effect could have important consequences for interpreting research and programme monitoring, but has rarely been assessed.

**Methods:**

A three-arm cluster-randomised trial of interventions to improve adherence to guidelines for the use of anti-malarial drugs was conducted in Tanzania. Patient interviews were conducted outside health facilities on two randomly-selected days per week. Health workers also routinely documented consultations in their ledgers. The Hawthorne effect was investigated by comparing routine data according to whether exit interviews had been conducted on three key indicators of malaria care. Adjusted logistic mixed-effects models were used, taking into account the dependencies within health facilities and calendar days.

**Results:**

Routine data were collected on 19,579 consultations in 18 facilities. The odds of having a malaria rapid diagnostic test (RDT) result reported were 11 % higher on days when exit surveys were conducted (adjusted odds ratio 95 % CI: 0.98-1.26, *p* = 0.097), 17 % lower for prescribing an anti-malarial drug to patients with a negative RDT result (0.56-1.23, *p* = 0.343), and 27 % lower for prescribing an anti-malarial when no RDT result was reported (0.53-1.00, *p* = 0.052). The effect varied with time, with a U-shaped association over the study period (*p* < 0.001). We also observed a higher number of consultations recorded on days when exit-interviews were conducted (adjusted mean difference = 2.03, *p* < 0.001).

**Conclusions:**

Although modest, there was some suggestion of better practice by health professionals on days when exit interviews were conducted. Researchers should be aware of the potential Hawthorne effect, and take into account assessment methods when generalising findings to the ‘real word’ setting. This effect is, however, likely to be context dependent, and further controlled evaluation across different settings should be conducted.

**Trial registration:**

ClinicalTrials.gov: NCT01292707. Registered on 29th January 2011.

**Electronic supplementary material:**

The online version of this article (doi:10.1186/s12879-016-1362-0) contains supplementary material, which is available to authorized users.

## Background

Observation of clinical consultations is an important and frequently used tool to assess the quality of care, but the process of observation may itself change how clinical staffs behave. This effect is generally referred to as "Hawthorne effect", from the industrial experiment of the 1920’s where worker’s productivity increased with every change made to the working conditions [1, 2]. Patient Exit Interviews involve an assessment of the patient as they leave a health facility. Typically a researcher will be stationed outside a health facility and will ask a series of questions, and possibly repeat a physical examination and/or clinical investigations, as patients leave the health facility. It is assumed that recall of the details of procedures in the health facility will be better when asked so soon after the consultation compared to retrospective alternatives.

If conducting such exit interviews affects the consultations, data collected through exit interviews could give a distorted picture of "real-life" consultations, with implications for program evaluation and implementation. The importance of the Hawthorne effect has been widely discussed in the literature [3–5] but has rarely been rigorously assessed [6]. In this paper, we document an assessment of the Hawthorne effect when exit interviews were conducted, as part of a randomised trial evaluating malaria diagnostic and treatment training in Northern Tanzania, by looking at routinely recorded health information. We sought to investigate the following hypotheses: i) documented malaria diagnostic and treatment practices differed when patient exit-interviews were conducted, ii) the difference also affected the recording of routine information not related to the trial outcomes, and iii) there were changes in the difference over time, as health workers became used to the exit interviews.

## Methods

### Trial setting

Data for this study were derived from the Targeting Artemisinin Combination Trial (TACT), a 3-arm cluster-randomised trial of different training interventions to improve the use of malaria rapid diagnostic tests (RDTs) among health workers in primary care facilities [7, 8]. The trial took place in two districts in northeast Tanzania in 2011–2012. This analysis was based on data collected in the Kilimanjaro region, a predominantly rural district with a relatively low malaria transmission and peak transmission seasons in April to June, and November to December. Participating primary care facilities (clusters) were randomised to one of three intervention arms. All prescribing health workers at the study facilities received the standard two-day national training on RDTs, where they were taught how to perform a RDT, and the recommended prescription practice (Artemisinin-based Combination Treatment (ACT) for positive test result, and no anti-malarial for negative test result) [9]. In addition health workers from the two intervention arms participated in three sessions of interactive training, aimed at reflecting on the change in practice and making it sustainable. The third arm also included the distribution of posters and patients leaflets to enhance demand for RDTs. The primary outcome of the trial was the proportion of patients with a non-severe, non-malarial illness being prescribed an approved antimalarial drug in a consultation for a new illness episode.

### Routine records

Health workers in primary care facilities in Tanzania are expected to keep a register of all their consultations. This Health Management Information System (HMIS, called MTUHA in Tanzania) includes a ledger where the health worker is supposed to record each patient's details, diagnoses and treatments. Each health worker has his own book. Records are aggregated and reported to the district level each month, with a summary of the number of patients seen by age (less than or over five years) and first diagnosis. As part of the trial, MTUHA records were modified to include information on fever and RDT result.

### Data collection

Trial outcomes were measured using patient exit surveys on 2 randomly-varied days per week throughout the trial. An exit survey interviewer was recruited from the nearby population using criteria of literacy and availability and given 2 days of training on site. On each day of the exit survey the interviewer notified the health staff of their presence. Survey dates changed occasionally from the initial schedule, due to practical issues such as weather conditions or interviewer availability.

The dispensary MTUHA register was inspected at the end of each survey day to extract the RDT result for each patient (identified on the basis of name, age and order seen), which served as a secondary source to validate exit interview information. Each dispensary was visited every four to six weeks by a research assistant to check on RDT and other essential supplies and to take a photograph of pages in the clinic register since the last visit. Health workers were informed of this and were told the RDT result was to be extracted.

A sample of the register data photographs were selected for data entry. Samples were taken from one of the trial region (Kilimanjaro), from three pre-defined time periods of two to three months, to represent the beginning middle and end of the one-year trial. The MTUHA data needed for this study were single-entered into MS Access (Microsoft Corp, Redmond VA).

Data from the Monday before the first exit survey and the Friday after the last survey (for each health facility) were included in this analysis.

### Measures definition

The main factor of interest, exit survey interview, was defined by at least one record in the TACT exit survey interview database for a given day, for the respective health facilities. We thereafter use the term Hawthorne effect to refer to the differences in indicators on survey compared to non-survey days. The indicators compared came from the MUTHA register completed by the health worker. Our three primary indicators were defined a priori as follow: i) having an RDT result reported, ii) whether an antimalarial drug prescription was reported for patients with a negative reported RDT result, and iii) whether an antimalarial drug prescription was reported for patients without a reported RDT result. Although ACT was the recommended treatment for malaria, the prescription of other antimalarials (likely due to stock outs of the ACTs) was documented and we included the prescription of any antimalarial in our analyses.

Other information from the MTUHA ledger used to asses completeness included the number of records per day and the patient’s age, gender, village of origin, previous attendance (during the same year, or for the same health problem in the last 2 weeks) and whether the patient contributed to the national health insurance scheme (subscriber type).

### Statistical analysis

A statistical analysis plan was written and published before initiating the analyses presented here [10]. Data and patients' characteristics were first reported descriptively, overall and by survey and non-survey days. General characteristics (distribution between health facilities, time period, day of the week (Monday-Friday), and patients’ characteristics) were compared between survey and non survey days. Differences were tested using Wald tests from appropriate hierarchical mixed-effect models [11] for each characteristic, taking into account clustering (non-independence) of data within each health facility, and within each day of data collection. Differences identified (days of the week and study period) were controlled for in the remaining analyses using fixed effects.

We investigated a possible Hawthorne effect on the general recording behaviour: number of records and completeness of general patient information (age, gender, village, previous attendance, subscriber type) were compared between survey and non survey days. The number of records per day was compared using a mixed-effect linear regression, with a random effect for clustering by health facilities. Completeness of information was compared using three-level random effect models to take into account the clustering by health facilities and by day of recording. When the mixed-effect models did not converge, simpler models with robust standard error allowing for clustering by health facility were used. The Hawthorne effect on our three primary outcomes was investigated in a similar way, comparing outcomes on survey to none survey days using a three-level random effect model. For each of the three models, we tested the absence of a differential effect by study arm by allowing for an interaction term between the Hawthorne effect and the two intervention arms combined, compared to the control arm.

Our third hypothesis was investigated by testing for an interaction between Hawthorne effect and time, first defined as the three study periods, then defined as a continuous variable in days from study initiation, and testing for a linear and quadratic interaction. To avoid issues of multiple comparisons it was decided a priori to test this hypothesis only on the RDT results recording outcome. Post hoc analyses were conducted to explore further this result, by plotting the change over smaller time periods, and by looking at the interaction between time and Hawthorne effect on the other two primary outcomes.

All statistical tests were two-sided and considered significant at the 5 % level. All statistical analyses were performed with Stata software version 13 (StataCorp, College Station, TX).

### Ethics

The nature and purpose of the trial was explained to participants and written informed consent was sought from heads of the facilities and all health workers. All attendees at study facilities were informed by leaflets and posters in each facility that basic data from their consultation might be recorded for research purposes. This was verbally repeated in the consultation and all subjects were free to refuse with no effect on the services offered. The study was approved by the Ethical Review Boards of the National Institute for Medical Research in Tanzania and the London School of Hygiene and Tropical Medicine (NIMRlHQ/R.8cNol. 11/24 and #5877 respectively). The trial was registered with clinicaltrials.gov (Identifier # NCT01292707). An independent data safety monitoring board monitored the trial and approved its’ overall statistical analysis plan.

## Results

### Data description

Eighteen health facilities contributed to the analysis. A majority (n = 16) were governmental, and two were funded by a mission. Each health facility typically comprised of three prescribing staff (range two to four), 75 % (39/52) of them above 45 years old, and 72 % (38/53) were female. Half (24/48) had worked in the facility for more than 10 years. There was an equal number (six) of health facilities from each of the three trial arms. Each facility had a median number of 85 days where MTUHA data were available, giving a total sample of 1,520 days for analysis, with 691 (45 %) on days when exit-survey interviews were conducted (Table 1).

**Table 1:** MTUHA data description

	Overall		Non-survey days		Survey days	
	n	%	n	%	n	%
	Median	(IQR)	Median	IQR	Median	(IQR)
Day characteristics	N = 1,520		N = 829		N = 691	
Study period						
* 1 – February-April 2011*	448	29.5 %	275	33.2 %	173	25.0 %
* 2 – June-July 2011*	539	35.5 %	293	35.3 %	246	35.6 %
* 3 – January-March 2012*	533	35.1 %	261	31.5 %	272	39.4 %
Number of days per health facility						
* Median (IQR)*	85	(77–95)	47	(40–53)	39	(36–41)
Number of patient records per day						
* Median (IQR)*	11	(7–16)	11	(7–16)	12	(8–17)
Patients characteristics	N = 19,579		N = 9,834		N = 9,745	
Age (years) (*N* = 19,340)						
* Median, IQR*	12	(3–36)	12	(3–35)	13	(3–38)
Gender (*N* = 19,530)						
* Male*	8,366	42.8 %	4,229	43.2 %	4,137	42.5 %
* Female*	11,164	57.2 %	5,569	56.8 %	5,595	57.5 %
Fever^a^ (*N* = 9,785)						
* Yes*	4,521	46.2 %	1,803	41.5 %	2,718	50 %
Diagnostic and treatment	N = 19,579		N = 9,834		N = 9,745	
RDT recorded						
* Yes*	3,821	19.5 %	1,811	18.4 %	2,010	20.6 %
RDT result (*n* = 3,821)						
* Positive*	199	5.2 %	90	5.0 %	109	5.4 %
* Negative*	3,622	94.8 %	1,721	95.0 %	1,901	94.6 %
AM prescription to RDT positive (*n* = 199)						
* No AM prescription recorded*	52	26.1 %	21	23.3 %	31	28.4 %
*ALu*	85	42.7 %	46	51.1 %	39	35.8 %
*Other AM*	62	31.2 %	23	25.6 %	39	35.8 %
AM prescribed to RDT negative (*n* = 3,622)						
* No AM prescription recorded*	3,294	90.9 %	1,553	90.2 %	1,741	91.6 %
* ALu*	275	7.6 %	148	8.6 %	127	6.7 %
* Other AM*	53	1.5 %	20	1.2 %	33	1.7 %
AM prescribed without RDT result (*n* = 15,758)						
* No AM prescription recorded*	15,366	97.5 %	7,813	97.4 %	7,553	97.6 %
* ALu*	275	1.8 %	160	2.0 %	115	1.5 %
* Other AM*	117	0.7 %	50	0.6 %	67	0.9 %

Frequencies reported next to characteristics when different from total

^a^Presence of fever was not part of routine data collection and was not always collected

*MTUHA* Mfumo wa Taarifa za Huduma za Afya (health management information system), *IQR* 1st and 3rd quartile, *ALu* artemether-lumefantrine, *AM* antimalarial drug

With a median of 11 consultation records per day, a total of 19,579 consultation records were available. A majority of patients were female (57 %), the median age was 12 years with 32 % below five years (Table 1). Fever was reported in 46 % of the consultations.

Table 1 shows details of malaria diagnostic testing and treatment reported in the MTUHA records. RDT results were reported in 20 % of consultations, with 5.2 % reported as positive. When restricted to patients where fever was documented (not reported in table) the proportion with a RDT result was 57 % (2,585/4,521) and 6.8 % (177/2,585) of those were positive. An antimalarial drug prescription was reported in 4.4 % (867/19,579) of consultations. The main antimalarial treatment prescribed was artemether/lumefantrine (ALu) (635/867, 73 %).

### Characteristics of days with and without exit-surveys

Some characteristics differed between days when surveys were conducted and those when no exit survey was done. The proportion of observed survey days per health facility ranged from 36 % to 71 % (*p*-value = 0.03). There was also a difference by time period (*p* < 0.001), with a lower proportion of data from surveyed days in the first study period (39 %, vs. 46 % and 51 % in the second and third periods respectively). Survey days were also associated with the days of the week (*p* < 0.001), with 11 % of surveys taking place on a Thursday, and 26 % on a Friday. The distribution of patients’ gender and age did not differ significantly between survey and non-survey days (*p* = 0.50 and *p* = 0.11, respectively).

### Hawthorne effect on general recording

Table 2 shows the median number of consultations per day was 11 on non-survey days and 12 and survey days. Adjusting for time period and day of the week, the difference was significant, with an average of 2.03 more consultations recorded on survey days (*p* < 0.001). The information recorded also differed on days when exit-surveys were conducted. Although age and gender were rarely missing, there was a possible association with more complete recording on survey days. Recording of village of origin, previous attendance, and subscriber type appeared to differ with surveyed days, although the direction of the difference was not consistent. On survey days, previous attendance appeared, although not significantly, less likely to be missing (Odds Ratio (OR) =0.54, *p* = 0.103), whereas village of origin and subscriber’s type were more likely to be missing (OR = 1.65, *p* = 0.01, and OR = 1.92, *p* < 0.001, respectively).

**Table 2:** Hawthorne effect on data recording and malaria practice

	Non-survey days		Survey days		Adjusted comparison^a^		
Number of consultations per day					*Difference*	*95 % CI*	*p*
* Mean (SD)*	11.9	(7.3)	14.1	(10.3)	2.03	1.20- 2.86	<0.001
* Median (IQR)*	11	(7–16)	12	(8–17)			
Missing MTUHA information	*n*	*%*	*n*	*%*	*OR*	*95 % CI*	*p*
Age	133	1.4 %	106	1.1 %	0.65	0.46-0.91	0.011
Gender	36	0.4 %	13	0.1 %	0.20^b^	0.05-0.86^b^	0.031^b^
Village of origin	5,937	60.4 %	6,919	71.0 %	1.65^c^	1.13-2.40^c^	0.010^c^
Previous attendance	1,604	16.3 %	2,127	21.8 %	0.54	0.26-1.13	0.103
Subscriber type	4,152	42.2 %	5,785	59.4 %	1.92^c^	1.38-2.67^c^	<0.001^c^
Malaria diagnostic and treatment (primary outcomes)	*n/N*	*%*	*n/N*	*%*	*OR*	*95 % CI*	*p*
RDT result recorded	1,811/9,834	18.4 %	2,010/9,745	20.6 %	1.11	0.98-1.26	0.097
AM prescription with a negative RDT	168/1,721	9.8 %	160/1,901	8.4 %	0.83	0.56-1.23	0.343
AM prescription without a RDT result	210/8,023	2.6 %	182/7,735	2.4 %	0.73	0.53-1.00	0.052

^a^Comparison of survey days to non-survey days, from mixed-effect logistic regression, adjusted for day of the week (Monday-Friday) and study period. Analyses based on three-level hierarchical models (with health facility and day of data collection as random effects), except for number of consultations per day, based on a two-level hierarchical model (health facility as random effect)

^b^Unadjusted, due to sparse data

^c^One-level logistic model, with robust standard errors for health facility clustering, due convergence failure for the hierarchical model

*RDT* Rapid Diagnostic Test, *AM* Antimalarial treatment, *SD* Standard deviation, *OR* Odds Ratio, *CI* Confidence Interval

### Hawthorne effect on malaria diagnostic and treatment practice

The comparison of the three primary outcomes between days with and without exit-surveys is reported in Table 2. After adjustment for time period and day of the week, all estimates suggested better practice on survey days, although none were statistically significant (*p* ≥ 0.052). There was a small non-significant difference for more RDTs being recorded on survey days (OR = 1.11, 95 % Confidence Interval (CI): 0.98-1.26, *p* = 0.097). The odds of having an antimalarial drug prescribed with a negative RDT result did not significantly differ, with 17 % lower odds on survey days (OR = 0.83, 95 % CI: 0.56-1.23, *p* = 0.343). Prescription of antimalarial when no RDT result was reported was borderline significantly lower on survey days, with 27 % lower odds (OR = 0.73, 95CI: 0.53-1.00, *p* = 0.052).

There was no indication of effect modification between trial arms (significance of interaction term *p* = 0.805, *p* = 0.800, *p* = 0.604, for the three outcomes, respectively).

### Change in Hawthorne effect over time

**Fig. 1:**
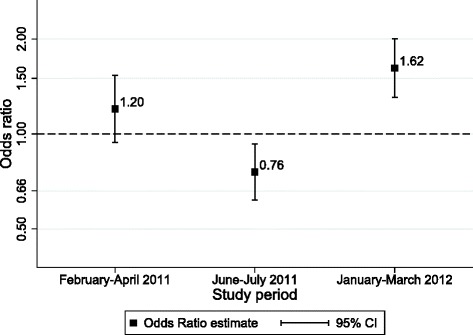
Hawthorne effect on reporting a RDT result, by study period. Odds ratio of reporting a RDT result for survey days compared to non-survey days. Estimates from a three-level hierarchical model (with health facility and calendar day as random effects) adjusted for day of the week, and stratified by study period. RDT = Rapid diagnostic test, *CI* Confidence Interval

We investigated whether the difference between survey and non survey days appeared to change over time. There was significant heterogeneity of the Hawthorne effect on RDT recording by period, with lower rates of RDT recording on survey days during the second period (OR = 0.76) than in the first and third periods (OR = 1.20 and 1.62, respectively) (Fig. 1). The test for interaction was significant (*p* < 0.001). When the Hawthorne effect was modelled by a linear and quadratic term for time effect, the quadratic term was significant. The odds ratio for the association between survey days and RDT recording increased of 0.3 % for every 100 squared days (OR = 1.003, *p* < 0.001).

To explore further this result, two post hoc analyses were conducted. The first one was to plot the Hawthorne effect by smaller time periods to see in more details the change over time. The quadratic shape of the change in Hawthorne effect remained, with higher effect at either ends of the study period (see Additional file 1). The second post hoc analysis explored the interaction for the two other primary outcomes. None of them showed evidence for heterogeneity of Hawthorne effect by study period (see Additional file 1), with the interaction terms not being significant (*p* = 0.43 for antimalarial drug prescription with a negative RDT, and *p* = 0.55 without a RDT result). The suggestion of a possible quadratic effect remained but confidence intervals were wide. In all cases, none of the three outcomes suggested a consistent reduction over time in the trend towards a Hawthorne effect.

## Discussion

We assessed indicators of case management, which were the subject of the research study and might therefore have been influenced when health staffs were under observation. This study did not find strong evidence that the presence of the exit survey altered the prescribing behaviour of health staff.

There is an increasing need to capture and monitor the performance of health staff in resource poor countries as investments in health services increases and the tasks expected of health staff become more complex and diverse. However there are relatively few established methodologies to capture the content of the consultation in primary care settings. One can review routine documentation of the consultation, although the reliability of self-reported practices is uncertain [12]. A commonly used alternative is to observe the consultation directly [13, 14]. This may be complemented by a repeat consultation by an “expert” immediately after the consultation of interest [15, 16]. These methods have a variety of potential limitations including the cost and practicality of having qualified health professionals to observe or repeat a consultation, and the strong influence that a peer observation may have on health workers. The patient exit survey is an interesting alternative [17–19], as it might reduce errors associated with inaccurate completion of routine records and minimise patient recall by asking about the content of the consultation immediately after its completion.

### The Hawthorne effect

All of these methods have the potential to alter the behaviour of health workers by creating anxiety, raising awareness from the novelty of the situation, or a desire to satisfy the expectation of the researchers. This ‘observer effect’ is generally referred to as the Hawthorne effect after the studies conducted in the Hawthorne electronics factory in the late 1920’s in Michigan, USA [1, 2]. Although definitions vary widely, it usually relates to the difference in someone’s behaviour when aware they are participating in research, or under scrutiny, as opposed to their behaviour in a more ‘natural’ setting. Rigorous evaluation of this effect is however limited [6], possibly explained by the complexity of this context-specific and multi-components concept, and also the challenges of measuring it without inducing it. Some studies have looked at the effect of direct observation on medical consultations [20–22]. Although the design was usually before and after and other factors could have influenced the result, they generally observed difference toward better practices when health workers were being observed. This study is, to our knowledge, the first evaluation of the Hawthorne effect when conducting patient exit interviews.

### Discussion of findings

Our primary results found no strong statistical evidence of important differences in clinical practice on days when exit surveys were conducted, but the differences we found were all in the direction of improved clinical practice on days when exit interviews were performed. The point estimates of effect size are modest, lying between 0.73 and 1.11, but with a lower confidence interval extending down to 0.53 for one of the outcomes. These results have implications for the interpretation of data captured through exit interviews and should be kept in mind when extrapolating data from exit surveys to “real world” practices. In the case of the TACT trial for example, the proportion of patients “appropriately treated” captured using exit interviews, could be an over-estimate.

The efficacy estimates could also be affected if the extent of the Hawthorne effect differed across trial arms, but this was not suggested by our analysis. All methods to assess case management have limitations [23] and the most complete overall picture is likely to result from triangulation of the results from a variety of methods.

It is pertinent to consider why the Hawthorne effect comes about. It is possible that participants become more attentive to their whole work routine, even for aspects of care which are not under scrutiny. On the other hand, by trying to excel in the practice being assessed, health workers may neglect other aspects of care. In our study we found suggestions of better record-keeping in the MTUHA book on the days where exit-surveys were conducted. This could suggest that consultations were more systematically recorded on the days where an external observer was present. There were also some indications of differences regarding completeness of other MTUHA information; however these results are to be interpreted with caution as the pattern of completion of some of the information remained unclear, and an appropriate statistical model could not always be performed.

The last hypothesis explored in this paper was the change in Hawthorne effect over time. The initial assumption was that the novelty effect may tend to reduce over time, as participant become used to being observed, and their ‘natural’ behaviour would return and dominate the observation-conditioned behaviour. The change in Hawthorne effect over time on our primary outcome (RDT uptake) was not as expected, as significant decrease, and then increase, in observer effect was observed (Fig. 1). In the second period of the study, health workers were significantly less likely to report an RDT result on survey days, for reasons which remain unclear. One hypothesis was that it could be related to the regular visits by research team to check supplies, after which health workers could have been more motivated to demonstrate good performance (even if this was not the aim of these visits). However visits were regular and do not seem to explain the curvilinear pattern. Seasonal variations in malaria transmission rates did not seem to explain the pattern either. More importantly, however, we did not find any suggestion of a reduction in the Hawthorne effect over time, on any of the three outcomes. Although it is often assumed than any Hawthorne effect would reduce over time we did not find any evidence of this here, and no such effect was actually evident in the original Hawthorne studies [24].

Some other interesting secondary findings include that no evidence was found for differences in Hawthorne effect between trial arms, which did not support that health workers in the intervention arms paid more attention to their practice on days when trial outcomes where measured, in order to satisfy the wishes of the investigators [25]. Another issue arising is the difficulty of working with routine data, particularly when coming from a handwritten book, then transferred into a database via photographs. Not all book pages could be recorded, and some patterns of information availability were surprising, for example the recording of the “village of origin” was completely missing on some days, and completely recorded on some other, without a clear explanation (such as different health workers, or variations in book format or workload). Electronic routine data recording could facilitate access and improve consistency, and recently introduced integrated systems of RDT reading and recording may also offer useful benefits [26].

### Generalisability

It seems likely that the Hawthorne effect is sensitive to the context of the study and our findings may not apply to other settings or methodologies. Our study was conducted in health facilities participating in a randomised trial, in one region of Tanzania, representing a very specific context. However use of exit surveys is common and the findings have some wider implications. There are clearly some specific conditions that are likely to modify the Hawthorne effect and these include any situation where some level of reward or sanction could result from the result of the study, or at least where it is expected as such by the health worker. The perception of an exit survey conducted as part of a trial may well be different from one conducted as part of a national monitoring programme. In addition it seems that more intense observation such as might occur with a researcher actually observing the consultation or where the consultation is replicated by an expert could also be expected to result in modified behaviour and our results are unlikely to apply to these situations. Having the interviews performed by a trained non-health professional from the community, may have reduced the fear of judgment for the health workers.

### Limitations

The study has a number of limitations. Firstly there was some knowledge among health staff that their routine records would be reviewed, although they were informed that this would be primarily to document the RDT result. All staffs were reassured that the results of the study would only be accessible to research staff and that data on individual health facilities or staff would not be revealed to anyone outside of the research team and in particular to senior or supervisory staff of the health clinics. Nonetheless, the trial could have affected the feeling of “scrutiny”, and health workers may have paid more attention to their practice even on days when exit surveys were not conducted, which would have reduced the apparent Hawthorne effect. The second major limitation is the reliance on completion of basic records and assumption that what was written was a true reflection of what was done. This is an inherent limitation of any study that aims to capture health worker performance without access to information obtained from direct observation. However, the main interest of the study was to investigate whether exit-surveys resulted in systematic difference in recording - we should therefore speak more of differences in ‘recording’ than differences in actual ‘practice’. Data used for this analysis were based on single data entry of photographs of the MTUHA records, and may not reflect the exact content of the book. For example, instances were reported where data could not be entered because the photos could not be read. Across the study periods, the median number of health facilities with data available on any specific day was 15 (out of 18). Again, this should not influence the assessment of the Hawthorne effect results if this is independent of survey days, but could bias the results otherwise (e.g. if health worker paid more attention to readability on days where exit surveys were conducted). Because survey were conducted on two randomly selected days per week, this design controlled for potential differences, and allows us to attribute the observed difference to the exit interview itself. However the schedule was not always strictly followed (see methods) or other biases could have occurred. We indeed observed differences in survey rates between health facilities, study periods and days of the week. We controlled for these factors in our analysis, but other unmeasured factors could have differed between surveyed and non-survey days and biased our findings. Another consideration is that what is reported here may not be considered as the whole Hawthorne effect, which would capture any difference in behaviour within and outside the research context. Here we have been able to capture the effect of conducting exit-surveys, but if health workers behave differently in general (even on days not monitored) because of participating in a trial, this would not have been captured here.

## Conclusion

Exit surveys of primary care consultations using staff recruited from the nearby community may have a modest effect on the clinical practice observed. It is important to consider the possibility of a Hawthorne effect when evaluating health interventions or monitoring routine health service provision, and to consider the extent to which this may alter the point estimates generated.

## Additional file

Additional file 1:
**Change in Hawthorne effect over time, exploratory analyses.** (PDF 9 kb)
