# Supplementary material for: Monitoring patient care through health facility exit interviews: an assessment of the Hawthorne effect in a trial of adherence to malaria treatment guidelines in Tanzania
Source: BMC Infect Dis. 2016 Feb 3;16:59. doi: 10.1186/s12879-016-1362-0 (PMC4739341; doi:10.1186/s12879-016-1362-0)

Additional file 1 – Change in Hawthorne effect over time, exploratory analyses

Odds ratio = Ratio of odds of experiencing outcome for survey days compared to non-survey days. Estimates from a three-level hierarchical model (with health facility and calendar day as random effects) adjusted for day of the week, and stratified by study period. RDT= Rapid diagnostic test, CI=Confidence Interval

AF1.1 Hawthorne effect on reporting a RDT result, by study period and subperiod
Each study period was split in 3 subperiods of equal sample size.

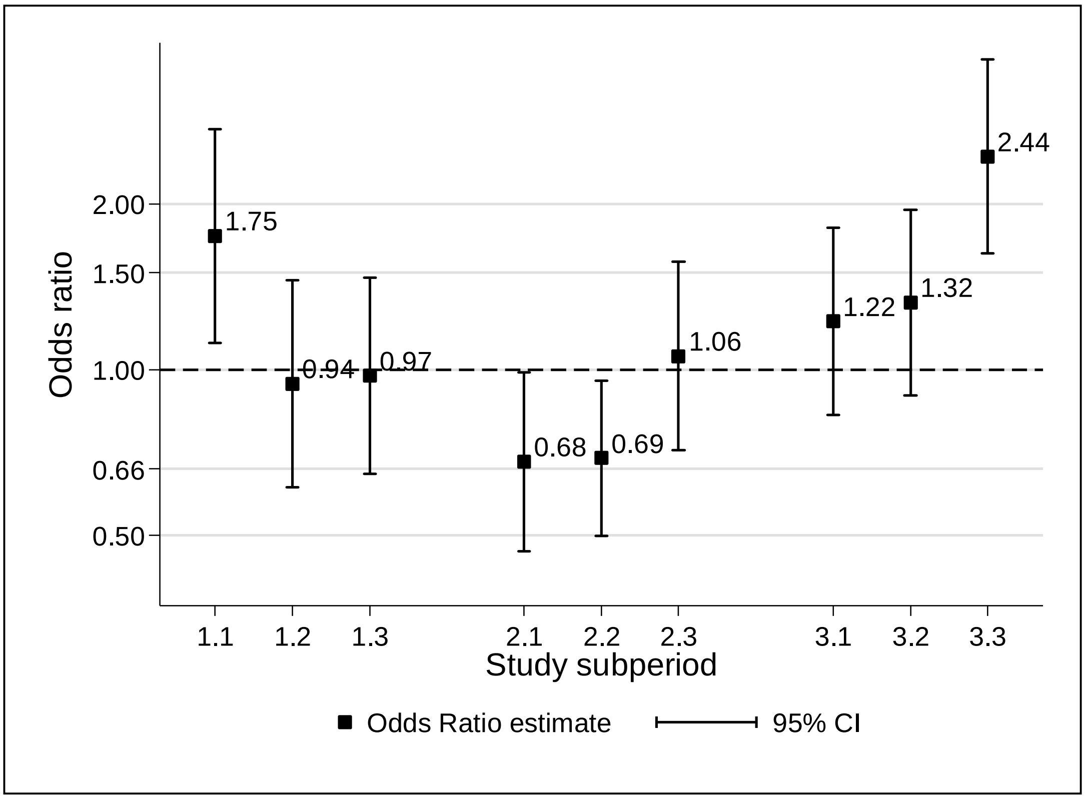

AF1.2 Hawthorne effect on antimalarial drug prescription among patients with negative RDT result, by study period

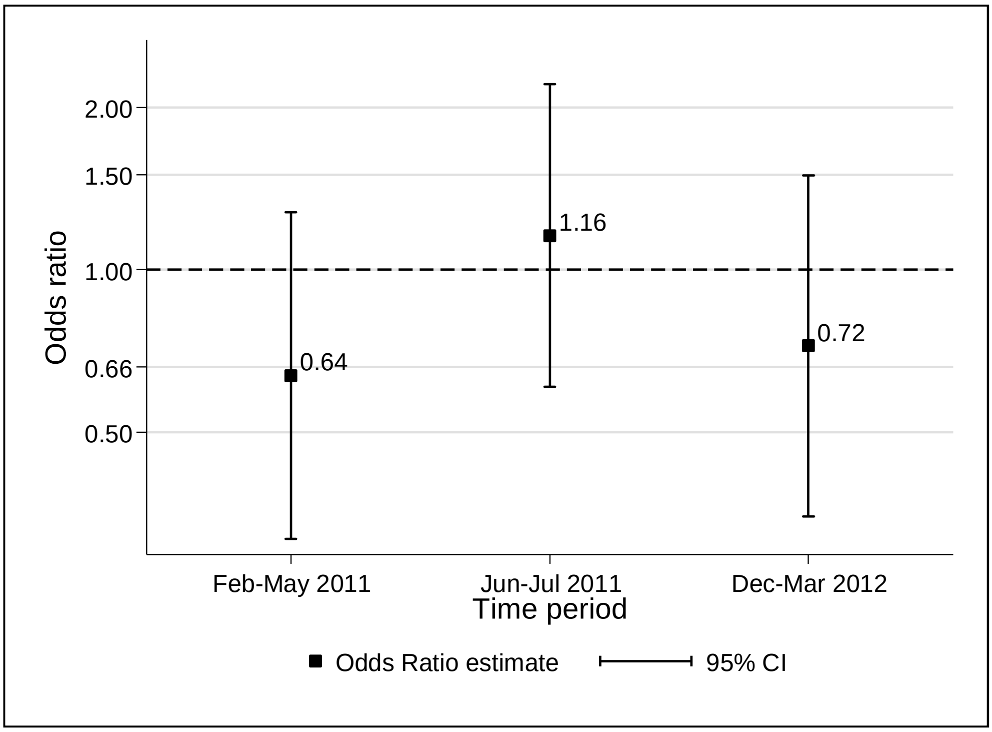

AF1.3 Hawthorne effect on antimalarial drug prescription among patients without RDT result, by study period

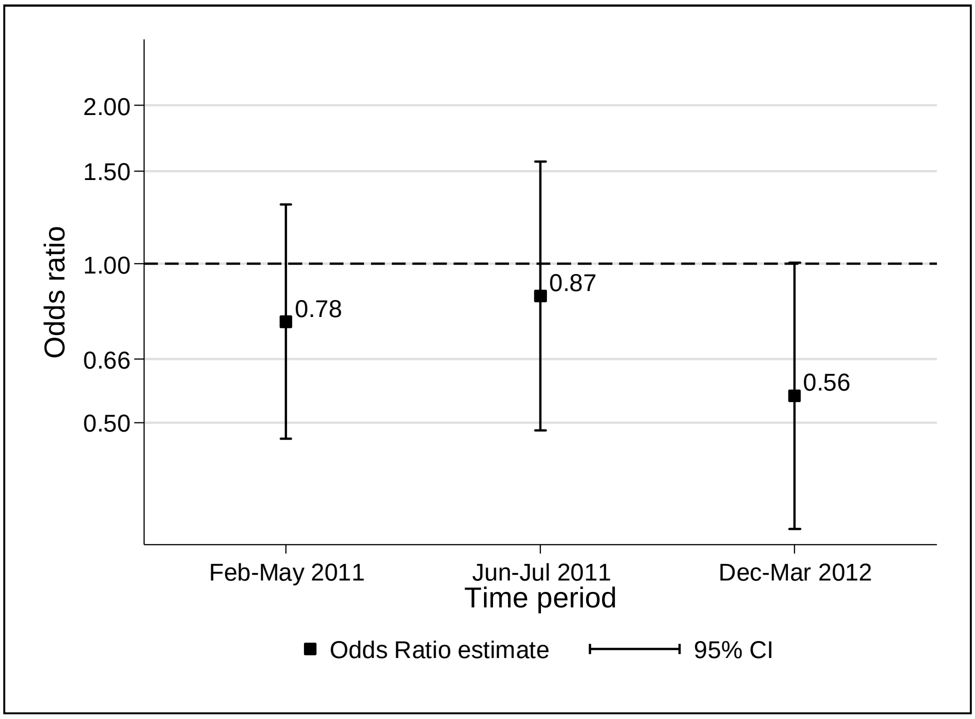

Supplement: Additional file 1: — Change in Hawthorne effect over time, exploratory analyses. (PDF 9 kb) [file 12879_2016_1362_MOESM1_ESM.pdf]
